# Identification of a key glioblastoma candidate gene, *FUBP3*, based on weighted gene co-expression network analysis

**DOI:** 10.1186/s12883-022-02661-x

**Published:** 2022-04-12

**Authors:** Jianmin Li, Zhao Zhang, Ke Guo, Shuhua Wu, Chong Guo, Xinfan Zhang, Zi Wang

**Affiliations:** 1 Department of Neurosurgery, Binzhou Medical University Hospital, Binzhou, Shandong Province People’s Republic of China; 2 Department of Pathology, Binzhou Medical University Hospital, Binzhou, Shandong Province China

**Keywords:** GBM, GEO, Immune-related gene, Molecular biomarkers, Survival analysis, WGCNA

## Abstract

**Background:**

Glioblastoma multiforme (GBM) is the most common aggressive malignant brain tumor. However, the molecular mechanism of glioblastoma formation is still poorly understood. To identify candidate genes that may be connected to glioma growth and development, weighted gene co-expression network analysis (WGCNA) was performed to construct a gene co-expression network between gene sets and clinical characteristics. We also explored the function of the key candidate gene.

**Methods:**

Two GBM datasets were selected from GEO Datasets. The R language was used to identify differentially expressed genes. WGCNA was performed to construct a gene co-expression network in the GEO glioblastoma samples. A custom Venn diagram website was used to find the intersecting genes. The GEPIA website was applied for survival analysis to determine the significant gene, *FUBP3*. OS, DSS, and PFI analyses, based on the UCSC Cancer Genomics Browser, were performed to verify the significance of *FUBP3*. Immunohistochemistry was performed to evaluate the expression of *FUBP3* in glioblastoma and adjacent normal tissue. KEGG and GO enrichment analyses were used to reveal possible functions of FUBP3. Microenvironment analysis was used to explore the relationship between FUBP3 and immune infiltration. Immunohistochemistry was performed to verify the results of the microenvironment analysis.

**Results:**

GSE70231 and GSE108474 were selected from GEO Datasets, then 715 and 694 differentially expressed genes (DEGs) from GSE70231 and GSE108474, respectively, were identified. We then performed weighted gene co-expression network analysis (WGCNA) and identified the most downregulated gene modules of GSE70231 and GSE108474, and 659 and 3915 module genes from GSE70231 and GSE108474, respectively, were selected. Five intersection genes (*FUBP3*, *DAD1*, *CLIC1*, *ABR*, and *DNM1*) were calculated by Venn diagram. *FUBP3* was then identified as the only significant gene by survival analysis using the GEPIA website. OS, DSS, and PFI analyses verified the significance of *FUBP3*. Immunohistochemical analysis revealed *FUBP3* expression in GBM and adjacent normal tissue. KEGG and GO analyses uncovered the possible function of *FUBP3* in GBM. Tumor microenvironment analysis showed that *FUBP3* may be connected to immune infiltration, and immunohistochemistry identified a positive correlation between immune cells (CD4 + T cells, CD8 + T cells, and macrophages) and *FUBP3*.

**Conclusion:**

*FUBP3* is associated with immune surveillance in GBM, indicating that it has a great impact on GBM development and progression. Therefore, interventions involving *FUBP3* and its regulatory pathway may be a new approach for GBM treatment.

## Introduction

Glioblastoma multiforme (GBM) is the most aggressive type of brain tumor, arising from the astrocytes, the neuroepithelial tissue group of the brain. GBM, also known as grade IV astrocytoma, is the most dangerous type of astrocytoma [1, 2]. The survival time for GBM patients is no more than 15 months [3]. Although surgery is generally used for GBM therapy [4], the cancer often relapses because of the lack of an effective prevention for GBM [5]. Effective biomarkers play a vital role in tumor treatment. Although there are many biomarkers closely related to GBM, including insulin-like growth factor 1 (IGF-1), vascular endothelial growth factor (VEGF), isocitrate dehydrogenase 1 (IDH1), and epidermal growth factor receptor (EGFR), no effective tumor markers have been discovered [6, 7]. Therefore, it is crucial to uncover appropriate and effective biomarkers to predict the prognosis for glioblastoma patients.

Over the last decade, gene set analysis has become the first choice for gaining insights into underlying complex biology of diseases through gene expression and gene association studies. It also reduces the complexity of statistical analysis and enhances the explanatory power of the obtained results [8]. In recent years, high-quality microarray techniques and high-throughput sequencing have been widely applied in clinical medicine and have created a new generation of molecular diagnostics based on DNA sequencing, RNA sequencing, and epigenetics [9]. Gene profiles can be obtained from public datasets such as Gene Expression Omnibus (GEO) and The Cancer Genome Atlas (TCGA), and the limitations between the combinations of different sample sizes and microarray platforms can be overcome by integrated bioinformatics methods. In order to find out the relationship among the selected genes, identification of key genes and gene modules responsible for a particular stress/condition, analysis of gene co-expression networks need to be carried out. Weighted Gene Co-expression Network Analysis (WGCNA) is a latest and popular technique used to decipher co-expression patterns among genes. The WGCNA approach typically deals with the identification of gene modules by using the gene expression levels that are highly correlated across samples [10]. And WGCNA has been successfully applied in many fields to identify candidate biomarkers and therapeutic targets [11].

In this study, we performed WGCNA for two RNA-Seq datasets derived from GEO and reconstructed gene co-expression networks, then we separately identified the most downregulated gene modules of the two datasets. A Venn diagram and GEPIA were used to find the intersecting genes of the two modules and further identify significant genes by survival analysis, and the significance of *FUBP3* was verified by Overall Survival (OS), Disease-Specific Survival (DSS), and Progression-Free Interval (PFI) analyses. We evaluated the expression of FUBP3 in GBM tissues and normal tissue adjacent to tumors by immunohistochemistry. We then considered the possible mechanism of action of *FUBP3* in GBM by KEGG and GO analyses and tumor microenvironment analysis based on TCGA tumor datasets, and immunohistochemistry identified a positive correlation between immune cells and *FUBP3*.

## Materials and methods

### WGCNA analysis of GEO

RNA sequence data from human glioblastoma samples were obtained from GEO datasets (http://www.ncbi.nlm.nih.gov/geo/). A microarray platform data table was used, annotating a series of matrix files with official gene symbols, and a gene expression matrix file was obtained for DEG analysis. The key search words were (glioma) AND “Homo sapiens”[porgn:__txid9606]. Finally, two datasets from the search results were included in our study, GSE70231 and GSE108474. These datasets contain glioblastoma and normal brain samples (GSE70231: GBM, *n* = 21; controls, *n* = 6, and GSE108474: GBM, *n* = 221; controls, *n* = 28) and were used to perform differential analysis. A series of matrix files and data sheets for the microarray platform were downloaded from the GEO website. The “limma” R package was used for DEG analysis. The DEG threshold was set as |log_2_FC|> 1, *P* < 0.05. The DEG sets for GSE70231 and GSE108474 were then selected (GEO70231_diff and GEO108474_diff).

The “WGCNA” R package was used to construct a co-expression network for all genes in the GBM and normal samples. Samples were used to construct Pearson’s correlation matrices, then the formula $a_{\;mn}=\left|c_{\;mn}\right|$  ^β^ (where ${a}_{mn}$ is adjacency between gene m and gene n, $c_{mn}$ is Pearson’s correlation, and β is the soft-power threshold) was used to construct a weighted adjacency matrix. This was then transformed into a TOM (topological overlap measure) matrix to evaluate the network connectivity, and a clustering dendrogram of the TOM matrix was constructed by average connected hierarchical clustering [12]. The threshold was set to 0.9 in GSE70231 and 0.95 in GSE108474. Then the *P*-values of the module eigengenes constructed by each sample and module gene were calculated by the WGCNA algorithm. The Pearson’s correlation coefficient was used to identify the connection between clinical traits and different modules, and the module with the highest Pearson’s correlation coefficient was selected for subsequent analysis. For our study, the most downregulated gene modules (GSE70231_turquoise and GSE108474_turquoise) in the GBM samples compared with healthy controls were selected.

### Key gene screening

A custom Venn diagram website (http://bioinformatics.psb.ugent.be/webtools/Venn/) was used to find the intersecting genes between GSE70231_diff, GSE108474_diff, GSE70231_turquoise, and GSE108474_turquoise.

### Verifying intersecting genes using GEPIA

The GEPIA website (http://gepia.cancer-pku.cn/) is a newly developed interactive web server for analyzing the RNA sequencing expression data of 9,736 tumors and 8,587 normal samples from the TCGA and GTEx projects, using a standard processing pipeline. In our study, GEPIA was used for survival analysis of the intersecting genes and to identify the significant key gene, *FUPB3*. First, we set the intersection gene used for normalizing in the “Gene” field, then we selected the OS survival method, chose GBM in the “Dataset Selection” field, and clicked “add” to build a dataset list in the “Datasets” field.

### Revalidating the key gene

The UCSC Cancer Genomics Browser (https://genome-cancer.ucsc.edu) offers interactive visualization and exploration of TCGA genomic, phenotypic, and clinical data, as produced by The Cancer Genome Atlas Research Network. The survival and survminer packages of R were used for overall survival(OS) analysis of *FUBP3* in GBM. The limma, survivor, and survminer R packages were used for the other survival analyses (DSS and PFI). The gene expression and clinical datasets were downloaded for 33 common types of cancer from UCSC based on TCGA datasets. Then *FUBP3* was used for three survival analyses, OS, DSS, and PFI, to revalidate the results found by the GEPIA website.

### Pathway analysis

The “pathway analysis” has been widely used in many ways including the analysis of Gene Ontology (GO) terms (also referred to as a “gene set”), physical interaction networks (e.g., protein–protein interactions), kinetic simulation of pathways, steady-state pathway analysis (e.g., flux-balance analysis), and in the inference of pathways from expression and sequence data. And in our study, we focused on methods that exploit pathway knowledge in public repositories such as gene ontology (GO) or Kyoto Encyclopedia of Genes and Genomes (KEGG) to identify pathways that may be affected in a condition by correlating information in at least one pathway knowledge base with gene expression patterns for the condition [13]. In recent years, the effective clustering of functional genes, mainly based on GO and KEGG, has been applied widely in DNA- and protein-related research [14]. The KEGG and GO databases can be downloaded from GSEA’s official website (https://www.gsea-msigdb.org/gsea/register.jsp). In our study, GO and KEGG analyses were used to reveal the possible function of *FUBP3* in GBM. The DEG functional analyses were performed using the clusterProfiler, org.Hs.eg.db, enrich-plot, and ggplot2 R packages, with *P* < 0.05 considered statistically significant.

### Microenvironment analysis

TIMER webserver (https://cistrome.shinyapps.io/timer/) is a comprehensive resource for systematic analysis of immune infiltrates across diverse cancer types. The abundances of six immune infiltrates (B cells, CD4 + T cells, CD8 + T cells, neutrophils, macrophages, and dendritic cells) were estimated by TIMER algorithm, and the gene module was selected to explore the correlation between *FUBP3* expression and the abundance of immune infiltrates.

### Immunohistochemical analysis

The human anti-FUBP3 monoclonal antibody came from Affinity Biosciences (Victoria, Australia), and the secondary antibodies, DAB color detection, enzyme-labeled goat anti-mouse/rabbit IgG polymer, mouse mAb assisted/induced T cell (CD4) antibody, rabbit mAb inhibitory/cytotoxic T cell (CD8) antibody, and mouse macrophage cell (CD68) antibody, were produced by Beijing Zhongshan Jinqiao Biotechnology. All the samples were selected from the Department of Pathology of the Affiliated Hospital of Binzhou Medical University and were double-blind rediagnosed by two senior pathologists. We selected 41 samples of human brain tissue from glioblastoma patients, from wax blocks that were serially sectioned, commonly dewaxed, rehydrated, and EDTA repaired. After intervention, we added H_2_O_2_ to the slices, after water jet and PBS washes. After serum blocking, human anti-FUBP3 monoclonal antibody (1:150), mouse mAb assisted/induced T cell (CD4) antibody, rabbit mAb inhibitory/cytotoxic T cell (CD8) antibody, and macrophage cell (CD68) antibody were added to both the glioblastoma tissue and normal tissue adjacent to the cancer, and incubated overnight at 4 °C. After water jet and PBS rinses the next day, the secondary antibody was added, then the slices were incubated for 30 min at 37 °C, and color developing agent was added to the glioblastoma tissue and the tissue adjacent to the cancer. Then, the tissue was stained restained with hematoxylin and differentiated by hydrochloric acid. Finally, they were washed with alcohol and left to dry. Cells expressing FUBP3 in the nucleus could be identified as the nucleus stained yellow or brownish-yellow, and CD68, CD4, and CD8 were expressed in the cytoplasm of microglia and T cells and appeared as brownish-yellow or light yellow colored granules. Ten independent fields of view images (400 ×) for each section were randomly chosen for image acquisition, and the five highest densities of FUBP3-expressed cells and immune cells were selected. The average value of the number of FUBP3-expressed cells and immune cells (CD4 + T cells, CD8 + T cells, and CD68 + macrophages) in each of the five fields were calculated. Samples with higher counts than average were classified as strong immune invasion, and samples with lower counts than average were classified as weak immune invasion. For the staining intensity, a score of 0 was given for cells without staining, 1 for light yellow, 2 for yellow–brown, and 3 for brown. For the number of positive cells, a score of 0 was given for ≤ 5%, 1 for 6%–25%, 2 for 26%–50%, 3 for 51%–75%, and 4 for > 75%. Then the two scores were multiplied, and a final score ≥ 4 was considered positive.

### Statistical analysis

SPSS 25.0 statistical software and R software (v 3.6.3) were used for statistical analysis. A statistical signifcance was identified between multiple groups by one-way ANOVA analysis of variance with the Tukey HSD test based on data in this study. The correlation analysis of protein expression was performed by spearman correlation analysis, and *P* < 0.05 was considered stastistically significant.

## Results

### Pre-processing of GSM RNA sequencing data

**Fig.1:**
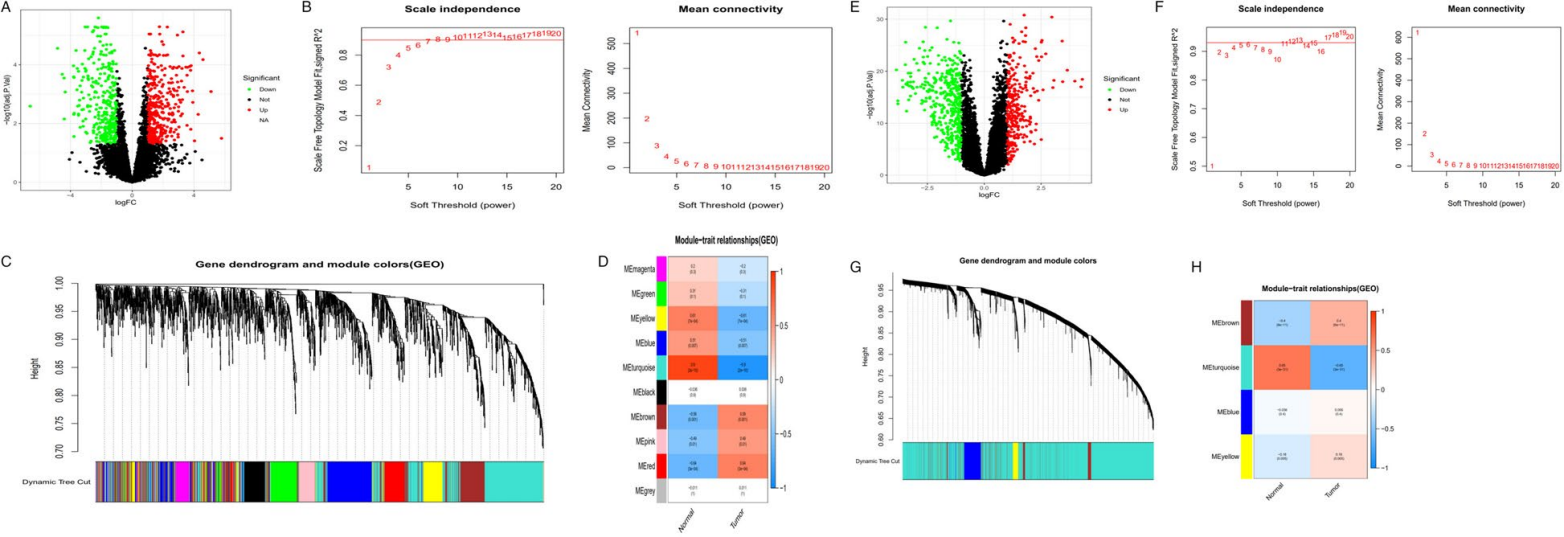
**A**-**D** Analysis of GSE70231 database: (**A**) The volcanic map of the genes that differ from the normal sample. **B** Determination of soft threshold (β) for weighted gene co-expression network analysis. **C** Gene cluster dendrogram. **D** Heat map of correlation between module features and clinical features. **E**–**H** Analysis of GSE108474 database: (The figures type are the same as database GSE70231)

Glioblastoma RNA-Seq data from GSE70231 and GSE108474 were downloaded from GEO datasets. Then we obtained a gene expression matrix file for DEG analysis (GBM patients compared with controls). The “limma” R package was used for the DEG analysis. The DEG threshold was set as |log_2_FC|> 1, *P* < 0.05. The GSE70231_diff dataset included 715 DEGs, consisting of 322 downregulated genes and 393 upregulated genes, while GSE108474_diff included 694 DEGs, consisting of 417 downregulated genes and 277 upregulated genes. These are shown in two volcano diagrams(Fig. 1: A, E).

### Weighted gene co-expression network analysis and module preservation

WGCNA was used to construct two gene co-expression networks for the glioblastoma and control samples of GSE70231 and GSE108474. The gene dendrogram constructed by dynamic tree was then identified, where each color represents one gene module. Then the module–trait relationships were determined. Finally, the most downregulated gene modules (GSE70231_turquoise and GSE108474_turquoise) from GSE70231 and GSE108474 were selected (Fig. 1: B-D, F–H).

### Screening co-expressed genes

**Fig. 2:**
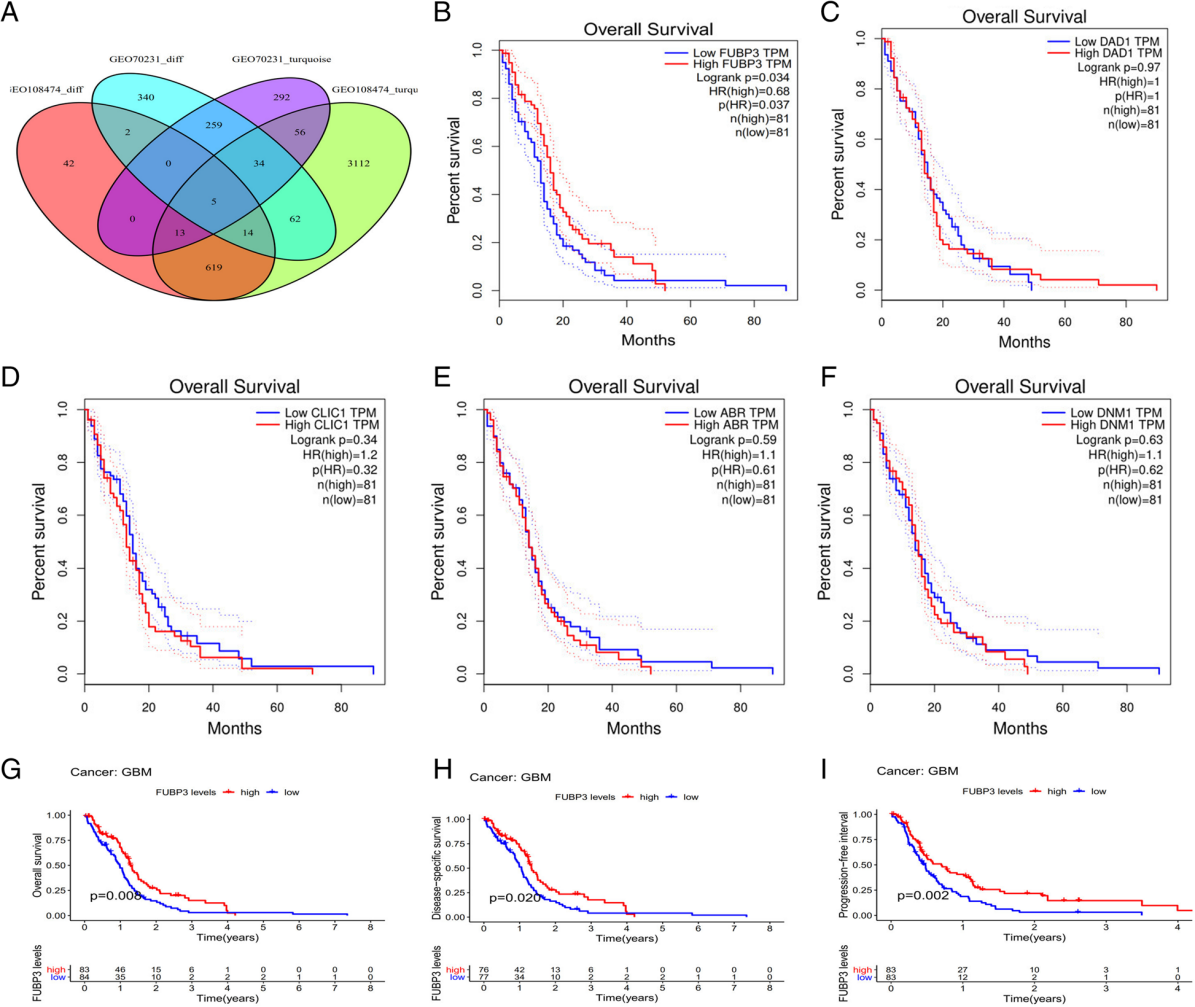
Venn: (**A**) DEGs were co expressed by four databases (GEO108474_diff,GEO70231_diff,GEO108474_turquoise,GEO70231_turquoise). **B**-**F**: The survival curves of continuous variables of 5 intersection genes (FUBP3,DAD1,CLIC1,ABR,DNM1). **G**-**I**: Three survival curves (OS, DSS, PFI) of continuous variables of FUBP3 in GBM

A custom Venn diagram website was used to calculate the intersection of the lists of elements in our study. We entered files (in plain text format) from four elements: GSE70231_diff, GSE108474_diff, GSE70231_turquoise, and GSE108474_turquoise, where GSE70231_diff contain 715 DEGs from GSE70231, GSE108474_diff contain 694 DEGs from GSE108474, GSE70231_turquoise contain the most downregulated gene module from GSE70231 and GSE108474_turquoise contain the most downregulated gene module from GSE108471 respectively based on WGCNA. Finally, five intersecting downregulated genes (*FUBP3*, *DAD1*, *CLIC1*, *ABR*, and *DNM1*) were found in common between the four datasets (Fig. 2: A).

### Verifying intersecting genes using GEPIA

The GEPIA website performs overall survival (OS), disease-free survival (DFS, also called relapse-free survival or RFS) analyses based on gene expression. This analysis selected *FUBP3* as the significant gene for OS (*P* < 0.05)(Fig. 2: B-F).

### Revalidating *FUBP3* based on the UCSC Cancer Genomics Browser

The results showed that when the overall survival was restricted to a particular level, the higher the *FUBP3* levels, the longer the GBM patients survived (*P* = 0.008). When the disease-specific survival was restricted to the same level, the higher the *FUBP3* levels, the longer the GBM patients survived (*P* = 0.02). When the progression-free interval was restricted to the same level, the higher the *FUBP3* levels, the longer the GBM patients survived (*P* = 0.002) (Fig. 2: G-I).

### Exploring the possible function of *FUBP3* in GBM based on KEGG and GO analyses

**Fig. 3:**
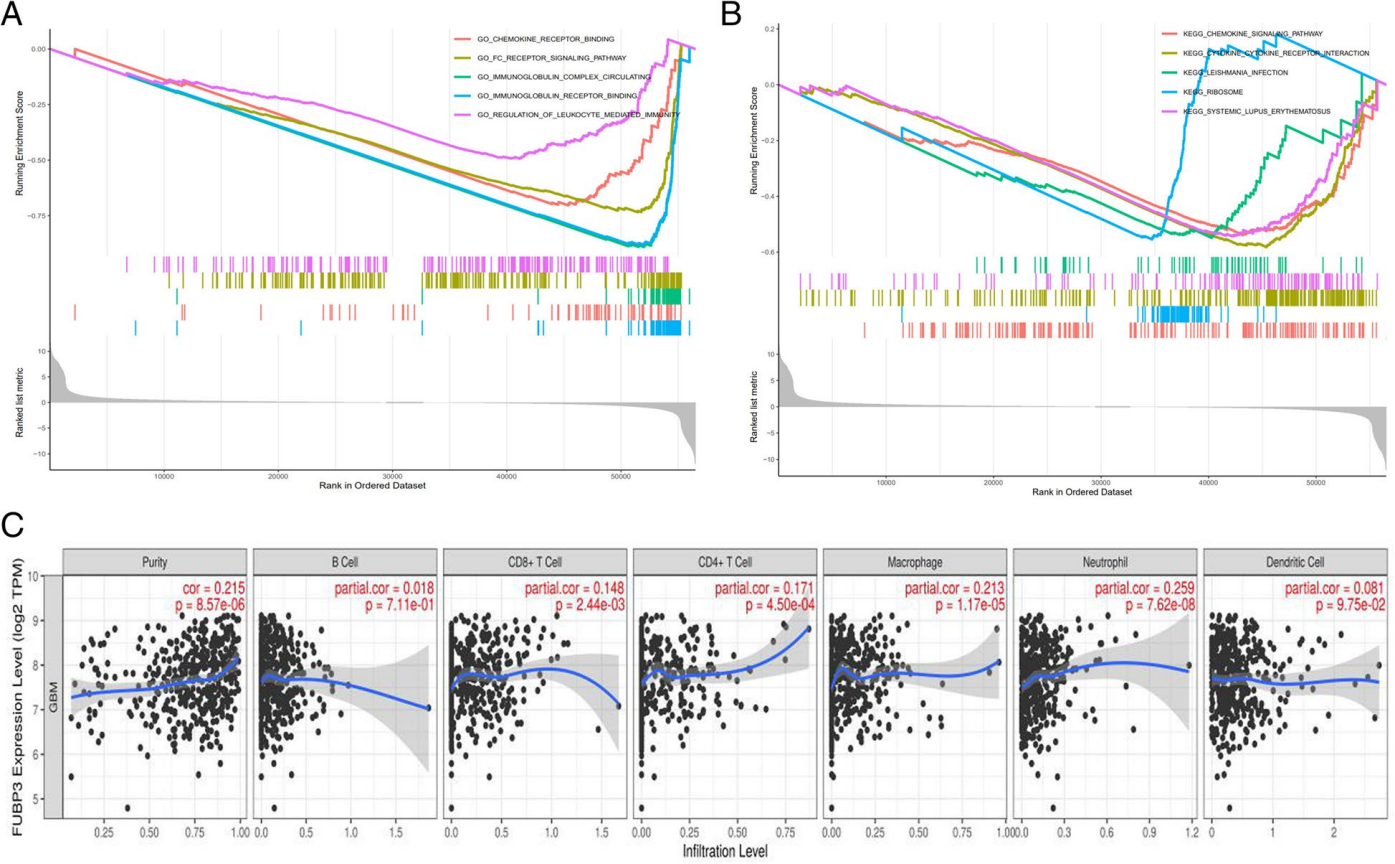
**A** Possible fuction of FUBP3 in GBM based on GO analysis. **B** Possible function of FUBP3 in GBM based on KEGG analysis. **C** Correlation between FUBP3 and immune cells

GO analysis showed that *FUBP3* may be mainly involved in chemokine receptor binding, the FC receptor signaling pathway, immunoglobulin complex circulating, immunoglobulin receptor binding, and regulation of leukocyte mediated immunity. KEGG analysis showed that *FUBP3* may be significantly associated with the chemokine signaling pathway, cytokine receptor interaction, leishmania infection, the ribosome, and systemic lupus erythematosus (Fig. 3: A-B).

### Tumor microenvironment analysis based on TIMER

The results showed that as the expression of *FUBP3* increased, the number of CD8 + T cells (cor = 0.148, *P* < 0.05), CD4 + T cells (cor = 0.171, *P* < 0.05), macrophages (cor = 0.213, *P* < 0.05), and neutrophils (cor = 0.259, *P* < 0.05) all increased. The relationships between *FUBP3* and B cells and dendritic cells were insignificant (both *P* < 0.05) (Fig. 3: C).

### Immunohistochemical verification of FUBP3 and immune-related cell expression

**Fig. 4:**
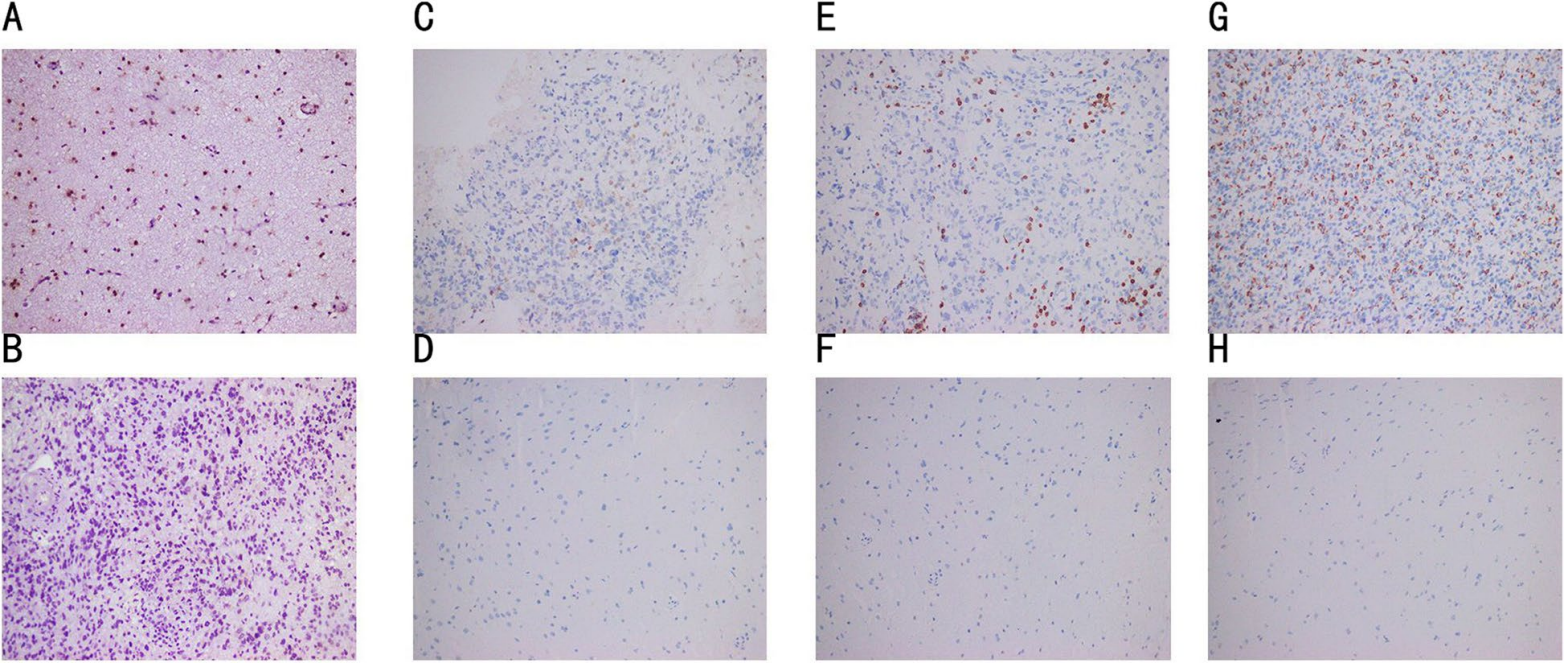
**A** The expression of FUBP3 in normal tissues adjacent to cancer. **B** The expression of FUBP3 in GBM tissues. **C** The expression of CD4 + T cell in GBM tissues. **D** The expression of CD4 + T cell in normal tissues adjacent to cancer. **E** The expression of CD8 + T cell in GBM tissues. **F** The expression of CD8 + T cell in normal tissues adjacent to cancer. **G** The expression of CD68 + macrophages in GBM tissues. **H** The expression of CD68 + macrophages in normal tissues adjacent to cancer

FUBP3 expression was identified by immunohistochemistry. FUBP3 was mainly expressed in the nucleus of the cell. In our study, FUBP3 was expressed in the normal tissue adjacent to the cancer and in the GBM samples to varying degrees. Through one-way analysis of variance comparison, compared with controls, the number of glioblastoma cells expressing FUBP3 was greater, but the color of the cell nuclei was lighter, meaning that the expression of FUBP3 in the cell nucleus of GBM tissues decreased (*P* < 0.05) (Fig. 4: A-B) (Table 1). The 41 GBM samples were divided into a high FUBP3 expression group and a low FUBP3 expression group based on the immunochemical assessment of the intensity of invasion. The infiltration intensities of CD4 + T cells, CD8 + T cells, and CD68 + macrophages in the FUBP3 high expression group were significantly stronger than those in the FUBP3 low expression group (*P* < 0.05) (Fig. 4: C-H) (Table 2).

**Table 1:** Expression of FUBP3 in GBM and normal tissue ajacent to cancer

	n	FUBP3		*P*
		+	-	
Normal	41	41	0	< 0.001*
Tumor	41	15	26	

*Notes*: *P < 0.05

*Abbreviation*: FUBP3: expression of FUBP3 in GBM and normal tissue ajacent to cancer

**Table 2:** Correlation between the expression of FUBP3 and immune cells of GBM tissues

GBM		n	FUBP3		r	*P*
			+	–		
CD4 +	+	16	13	3	0.742	< 0.001*
	–	25	2	23		
CD8 +	+	20	14	6	0.677	< 0.001*
	–	21	1	20		
CD68 +	+	17	13	4	0.697	< 0.001*
	–	24	2	22		

*Notes*: *P < 0.05

*Abbreviation*: Correlation between the expression of FUBP3 and immune cells(CD4 + , CD8 + , CD68 +) of GBM tissues

## Discussion

Glioblastoma (GBM) is the most common primary malignant brain tumor, accounting for 16% of all primary brain and central nervous system neoplasms [15]. Currently, the standard therapy for GBM includes surgical resection followed by timozolomide (TMZ) [16], but it is difficult to remove the tumor completely, so the surrounding brain tissue remains with tumor infiltration, leading to tumor progression or relapse [17]. Despite great effort, little progress has been made towards prolonged survival of GBM. Therefore, it is important to identify significant prognostic biomarkers, such as mutations in isocitrate dehydrogenase (*IDH*) and O6-methylguanine-methyltransferase (*MGMT*) promoter methylation, to improve the prognosis for GBM patients.

In this study, bioinformatics methods and clinical experiments were used to analyze the DEGs between GBM and normal samples to explore the key candidate genes. After pre-processing of the RNA sequencing data, 715 DEGs from GSE70231 were included, consisting of 322 downregulated and 393 upregulated genes, and 694 DEGs from GSE108474 were included, consisting of 417 downregulated and 277 upregulated genes. Then, WGCNA was performed to determine the modules of the most downregulated genes (GSE70231_turquoise and GSE108474_turquoise) from GSE70231 and GSE108474, to reveal the key candidate genes. A custom Venn diagram website was used to find the intersecting genes (*FUBP3*, *DAD1*, *CLIC1*, *ABR*, and *DNM1*). *FUBP3* was selected as the significant gene for OS (overall survival) by the GEPIA website. Then immunohistochemistry was used to identify the expression of FUBP3 in GBM and normal samples. The results showed that the expression of FUBP3 in the cell nuclei of GBM tissue decreased compared with that of the normal tissue adjacent to the tumor. The survival analyses (OS, DFS, and PFI) showed that when the survival rate was restricted at the same level, the higher the FUBP3 levels, the longer the GBM patients survived (*P* < 0.05). Then the possible function of FUBP3 based on GO and KEGG analyses were evaluated. GO analysis showed that *FUBP3* may be mainly involved in chemokine receptor binding, the FC receptor signaling pathway, immunoglobulin complex circulating, immunoglobulin receptor binding, and regulation of leukocyte mediated immunity. KEGG analysis showed that *FUBP3* may be significantly associated with the chemokine signaling pathway, cytokine receptor interaction, leishmania infection, the ribosome, and systemic lupus erythematosus. Tumor microenvironment analysis showed that *FUBP3* expression was positively correlated with the expression of CD8+ T cells, CD4+ T cells, and macrophages (*P* < 0.05). We further verified the expression of FUBP3 and immune cells by immunohistochemistry.

FBP3 (encoded by *FUBP3*) is a member of a mammalian three gene family of single-strand nucleic acid binding proteins, also including FBP2 (encoded by *KHSRP*) and FBP (encoded by *FUBP1*). The structure of the protein consists of four regular K homologous motifs that can recognize single-stranded RNA or DNA sequences [18]. The MYC transcription factor plays an important role in cell differentiation, growth, and senescence. Single-stranded DNA of the far upstream element (*FUSE*), 1.7 kb upstream of the major P2 promoter of the human *MYC* gene, is bound by FBP (FUSE binding protein) [19–21] , and the specific area p89/XPB/ERCC3 30 to 50 helicase/translocase of transcription factor II H (TFIIH) is stimulated [22–24]. As transcription increases, FIR(FUBP interference inhibitor) is recruited through DNA–protein and protein–protein interactions, and then FIR reduces TFIIH activity to a basic level to suppress transcription, maintaining the balance of the levels of MYC [25–28]. FUBP3 is closely related to many cancers. For instance, the FUBP3–c-Myc axis is activated to promote colorectal cancer progression [29], and FUBP3 is more frequently expressed in prostate and bladder cancer than in renal cancer. A positive relationship between the expression of FUBP3 and c-Myc is detectable [30]. Some studies have shown that increased expression of FUBP1 is a predictor of poor survival in human glioma [31]. Different members of the FUBP family have different functions in different cancers, and FUBP3 plays different roles in different tumors. Through intersection analysis of the general prognostic factors for *FUBP3*, we found that there was a positive correlation between *FUBP3* and the amount of infiltration of CD4+ T cells, CD8+ T cells, and macrophages in the GBM microenvironment. Therefore, we carried out further verification and found that the expression level of FUBP3 was lower in GBM samples than in normal tissue adjacent to the tumor, and the higher the expression level of FUBP3, the longer the GBM patients survived. The expression of FUBP3 was positively correlated with the expression of CD8+ T cells, CD4+ T cells, and macrophages in tumor tissue, based on immunohistochemistry. Cancer immunosurveillance has been recognized as a component of the general process of cancer immunoediting, which could be responsible for eliminating tumors [32]. However, the central nervous system (CNS) is thought to be exempt from the effects of the immune system. The brain has physical barriers for protection, and cells in the nervous system respond to inflammation and injury in unique ways [33]. CD8+ T cells constitute an important branch of adaptive immunity, contributing to the clearance of intracellular pathogens and providing long-term protection [34]. CD4+ T cell subsets have the ability to “dedifferentiate” given appropriate environmental signals, allowing individuals to respond to environmental stimuli in a context-dependent manner. A balance of CD4+ T cell subsets is critical to mount responses against pathogen challenges to prevent inappropriate activation, maintain tolerance, and participate in antitumor immune responses [35]. Tumor-associated macrophages (TAMs) represent the most abundant immune cells within the tumor microenvironment and have been associated with adverse outcomes in patients with different types of cancer [36]. There are two main macrophage phenotypes, M1 and M2. Classically activated M1 macrophages promote the antitumor immune response by modulating antigen presentation and secreting proinflammatory cytokines, while activated M2 macrophages play an immunosuppressive role [37]. The TAM phenotypes can be determined by antibodies against TAM-associated biomarkers, such as CD68 (macrophage marker), iNOS (M1 marker), and CD163 (M2 marker). Therefore, due to the blood-brain barrier the central nevrous system(CNS) has been recognized as an immune-free zone [38]. We speculated that FUBP3 was highly expressed in normal brain tissue to maintain the balance of immune cells. Cancer cells with mutated genes can produce mutated proteins that would not normally be produced, and these protiens can be recognized as foreign by the immune system, allowing macrophages to discover cancer cells [39]. In the FUBP3(+) GBM group, the expression could enhance the antigen presentation of CD68+ macrophages through specific molecular pathways (chemokine receptor binding and the FC receptor signaling pathway), stimulating the immune effects of CD4+ and CD8+ T cells to enhance the killing effect on tumor cells. Conversely, the immune monitoring effect is weakened and tumor prognosis is poor for the FUBP3-lacking GBM group.

Collectively, our results suggest that *FUBP3* in GBM is a potential predictor for the malignancy of the tumor. However, this result needs to be studied further in a larger group.

## Conclusion

In summary, the purpose of this study was to identify a key gene that may be relevant to the prediction and prognosis of GBM patients. A WGCNA approach with GBM RNA-Seq data was performed to find intersecting genes, and survival analysis was used to determine the significant key gene, *FUBP3*. We speculated that FUBP3 could accelerate the death of glioblastoma cells and increase the survival rate of patients by activating immune cells (CD4+ T cells, CD8+ T cells, and CD68+ macrophages). We found that *FUBP3* is a potential biomarker for the prediction, prognosis, and treatment of GBM. However, the function and specific pathway requires further study.

## Data Availability

RNA sequence data from human glioblastoma samples (GSE70231 and GSE108474) were obtained from GEO datasets (http://www.ncbi.nlm.nih.gov/geo/). Venn diagram website (http://bioinformatics.psb.ugent.be/webtools/Venn/). GEPIA website (http://gepia.cancer-pku.cn/). UCSC Cancer Genomics Browser (https://genome-cancer.ucsc.edu). GSEA’s official website (https://www.gsea-msigdb.org/gsea/register.jsp). TIMER webserver (https://cistrome.shinyapps.io/timer/).

## Abbreviations

GBM: Glioblastoma
WGCNA: Weighted gene co-expression network analysis
TCGA: The Cancer Genome Atlas
GEO: Gene Expression Omnibus
GO: Gene Ontology
KEGG: Kyoto Encyclopedia of Genes and Genomes
OS: Overall survival
DFF: Disease-specific survival
PFI: Progression-free interval
TIMER: Tumor IMmune Estimation Resource

## References

[1] Ohgaki H, Kleihues P (2005). Epidemiology and etiology of gliomas. Acta Neuropathol.

[2] Maher EA, Brennan C, Wen PY (2006). Marked genomic differences characterize primary and secondary glioblastoma subtypes and identify two distinct molecular and clinical secondary glioblastoma entities. Cancer Res.

[3] Young RM, Jamshidi A, Davis G, Sherman JH (2015). Current trends in the surgical management and treatment of adult glioblastoma. Ann Transl Med.

[4] Nam JY, de Groot JF (2017). Treatment of glioblastoma. J Oncol Pract.

[5] Gallego O (2015). Nonsurgical treatment of recurrent glioblastoma. Curr Oncol.

[6] Verhaak RG, Hoadley KA, Purdom E (2010). Integrated genomic analysis identifies clinically relevant subtypes of glioblastoma characterized by abnormalities in PDGFRA, IDH1, EGFR, and NF1. Cancer Cell.

[7] Cancer Genome Atlas Research Network (2008). Comprehensive genomic characterization defines human glioblastoma genes and core pathways [published correction appears in Nature. 2013 Feb 28;494(7438):506]. Nature.

[8] Das S, McClain CJ, Rai SN (2020). Fifteen years of gene set analysis for high-throughput genomic data: a review of statistical approaches and future challenges. Entropy (Basel).

[9] Head SR, Komori HK, LaMere SA (2014). Library construction for next-generation sequencing: overviews and challenges. Biotechniques.

[10] Das S, Meher PK, Rai A (2017). Statistical approaches for gene selection, hub gene identification and module interaction in gene co-expression network analysis: an application to aluminum stress in soybean (Glycine max L.). PLoS One.

[11] Langfelder P, Horvath S (2008). WGCNA: an R package for weighted correlation network analysis. BMC Bioinformatics.

[12] Chen S, Yang D, Lei C (2019). Identification of crucial genes in abdominal aortic aneurysm by WGCNA. PeerJ.

[13] Khatri P, Sirota M, Butte AJ (2012). Ten years of pathway analysis: current approaches and outstanding challenges. PLoS Comput Biol.

[14] Chen L, Zhang YH, Wang S, Zhang Y, Huang T, Cai YD (2017). Prediction and analysis of essential genes using the enrichments of gene ontology and KEGG pathways. PLoS One.

[15] Thakkar JP, Dolecek TA, Horbinski C (2014). Epidemiologic and molecular prognostic review of glioblastoma. Cancer Epidemiol Biomarkers Prev.

[16] National Comprehensive Cancer Network. (2015). Clinical Practice Guidelines in Oncology: Central nervous system cancers [v.1.2015]. Retrieved from https://www.nccn.org/professionals/ physician_gls/pdf/cns.pdf.

[17] Wilson TA, Karajannis MA, Harter DH (2014). Glioblastoma multiforme: State of the art and future therapeutics. Surg Neurol Int.

[18] Zhou W, Chung YJ, Parrilla Castellar ER (2016). Far upstream element binding protein plays a crucial role in embryonic development, hematopoiesis, and stabilizing Myc expression levels. Am J Pathol.

[19] Avigan MI, Strober B, Levens D (1990). A far upstream element stimulates c-myc expression in undifferentiated leukemia cells. J Biol Chem.

[20] Duncan R, Bazar L, Michelotti G (1994). A sequence-specific, single-strand binding protein activates the far upstream element of c-myc and defines a new DNA-binding motif. Genes Dev.

[21] Michelotti GA, Michelotti EF, Pullner A, Duncan RC, Eick D, Levens D (1996). Multiple single-stranded cis elements are associated with activated chromatin of the human c-myc gene in vivo. Mol Cell Biol.

[22] Liu J, Akoulitchev S, Weber A (2001). Defective interplay of activators and repressors with TFIH in xeroderma pigmentosum. Cell.

[23] Liu J, Kouzine F, Nie Z (2006). The FUSE/FBP/FIR/TFIIH system is a molecular machine programming a pulse of c-myc expression. EMBO J.

[24] Weber A, Liu J, Collins I, Levens D (2005). TFIIH operates through an expanded proximal promoter to fine-tune c-myc expression. Mol Cell Biol.

[25] Braddock DT, Louis JM, Baber JL, Levens D, Clore GM (2002). Structure and dynamics of KH domains from FBP bound to single-stranded DNA. Nature.

[26] Cukier CD, Hollingworth D, Martin SR, Kelly G, Díaz-Moreno I, Ramos A (2010). Molecular basis of FIR-mediated c-myc transcriptional control. Nat Struct Mol Biol.

[27] Crichlow GV, Zhou H, Hsiao HH (2008). Dimerization of FIR upon FUSE DNA binding suggests a mechanism of c-myc inhibition. EMBO J.

[28] Hsiao HH, Nath A, Lin CY, Folta-Stogniew EJ, Rhoades E, Braddock DT (2010). Quantitative characterization of the interactions among c-myc transcriptional regulators FUSE, FBP, and FIR. Biochemistry.

[29] Gao Q, Zhou R, Meng Y (2020). Long noncoding RNA CMPK2 promotes colorectal cancer progression by activating the FUBP3-c-Myc axis. Oncogene.

[30] Weber A, Kristiansen I, Johannsen M (2008). The FUSE binding proteins FBP1 and FBP3 are potential c-myc regulators in renal, but not in prostate and bladder cancer. BMC Cancer.

[31] Ding Z, Liu X, Liu Y (2015). Expression of far upstream element (FUSE) binding protein 1 in human glioma is correlated with c-Myc and cell proliferation. Mol Carcinog.

[32] Dunn GP, Bruce AT, Ikeda H, Old LJ, Schreiber RD (2002). Cancer immunoediting: from immunosurveillance to tumor escape. Nat Immunol.

[33] Gemma C (2010). Neuroimmunomodulation and aging. Aging Dis.

[34] Mittrücker HW, Visekruna A, Huber M (2014). Heterogeneity in the differentiation and function of CD8^+^ T cells. Arch Immunol Ther Exp (Warsz).

[35] Caza T, Landas S. Functional and Phenotypic Plasticity of CD4(+) T Cell Subsets. Biomed Res Int. 2015;2015:521957. 10.1155/2015/521957 PMC4637038 26583116

[36] Bindea G, Mlecnik B, Tosolini M (2013). Spatiotemporal dynamics of intratumoral immune cells reveal the immune landscape in human cancer. Immunity.

[37] Ostuni R, Kratochvill F, Murray PJ, Natoli G (2015). Macrophages and cancer: from mechanisms to therapeutic implications. Trends Immunol.

[38] Koelzer VH, Canonica K, Dawson H (2015). Phenotyping of tumor-associated macrophages in colorectal cancer: impact on single cell invasion (tumor budding) and clinicopathological outcome. Oncoimmunology.

[39] Chen Z, Hambardzumyan D (2018). Immune Microenvironment in Glioblastoma Subtypes. Front Immunol.

